# Diagnostic- and age-based measurement invariance of irritability in youths

**DOI:** 10.1007/s00787-026-03037-z

**Published:** 2026-05-09

**Authors:** Jillian M. Russo, Alessandro S. De Nadai, Kathryn Parker, Julianne G. Wilner, Angela Salisbury, Nathaniel P. Shogren, Emily G. Arnott, Sydney M. Tucker, Daniel P. Dickstein

**Affiliations:** 1 Pediatric Mood, Imaging, and NeuroDevelopment (PediMIND) Program, McLean Hospital, 115 Mill St, Belmont, MA 02478 USA; 2 McLean Hospital Simches Division of Child and Adolescent Psychiatry, Harvard Medical School, Belmont, USA

**Keywords:** Irritability, Transdiagnostic, Child, Adolescent

## Abstract

Irritability is one of the most common transdiagnostic symptoms. It spans across mood, anxiety, and other disorder classes and is listed as a top problem in outpatient settings. Irritable mood is a symptom for at least 10 disorders in the DSM-5, and it is also developmentally normal across the lifespan. Given this background, we investigated how irritability maps across disorders and ages of children and adolescents. Participants (*N* = 186) ages 8–16 were enrolled in two ongoing IRB-approved studies investigating the nature of irritability in a transdiagnostic sample of children and adolescents. Participants completed both a child and parent version of the Affective Reactivity Index (ARI-C and ARI-P, respectively) and the Kiddie Schedule for Affective Disorders and Schizophrenia (KSADS) was administered by a trained clinician. Multiple indicators multiple causes (MIMIC) modeling was used to assess for differential item functioning among items in the ARI-P/C compared to diagnoses of major depressive disorder (MDD), generalized anxiety disorder (GAD), oppositional defiant disorder (ODD), and attention-deficit hyperactive disorder (ADHD). ARI-P showed differential item functioning (DIF) with MDD, GAD, and ADHD and participant age. ARI-C showed DIF with ADHD. However, the DIF effect sizes were small. These results indicate child and parent reports of child irritability manifest similarly regardless of specific diagnosis or age. Further understanding of how irritability presents itself across disorders and the lifespan can allow for improved assessment and treatment.

**Supplementary Information:**

The online version contains supplementary material available at https://doi.org/10.1007/s00787-026-03037-z.

## Introduction

Irritability is among the most common and impairing symptoms in child mental health. Specifically, irritability is a top complaint by over 58% of children and parents seeking mental health services [1]. This is higher than those who identify symptoms of depression, anxiety, and inattention and/or hyperactivity as a presenting problem [1]. It is associated with over 10 different DSM-5 diagnoses [2]. Irritability is an explicit diagnostic criterion for a manic episode and, in children, a major depressive episode in major depressive disorder (MDD) [2]. Additionally, it is listed among the diagnostic criteria for other disorders such as generalized anxiety disorder (GAD) and posttraumatic stress disorder (PTSD) [2]. In DSM-5, irritability is one of three components of oppositional defiant disorder (ODD) and the defining criteria for disruptive mood dysregulation disorder (DMDD) [2, 3]. While not a diagnostic criterion, irritability is commonly associated with other disorders such as attention-deficit hyperactivity disorder (ADHD) and autism spectrum disorder (ASD) [4]. Given its ubiquitousness, a greater understanding of the relationship among irritability, psychiatric diagnoses, and impairment in children and adolescents is important.

Many studies have examined irritability in specific disorders. As irritability is listed as mood criterion for both a depressive and manic episode, researchers have investigated how commonly it presents in children with MDD and bipolar disorder (BD). Studies have found that approximately one third of youth with MDD present with both depressed mood and irritability while 10% of youth with BD presented with irritability only at baseline [5, 6]. In a longitudinal follow-up study conducted by Hunt and colleagues [7], they found that the individuals with BD who presented with only irritability at baseline were more likely to display depressive symptoms at follow-up. Additionally, irritability only at baseline was not predictive of its severity across the course of illness [7]. Irritability is also listed as a diagnostic symptom for GAD. Comer and colleagues [8] found that approximately 90% of youth seeking treatment for GAD endorsed persistent irritability. In studying children with ODD, irritability is a core component of the disorder, along with headstrong and vindictive symptoms [3, 9]. For children with DMDD, chronic irritability must be present for at least a year. In community samples, 0.8%−3.3% of children met diagnostic criteria for DMDD [10]. In summary, research has shown that irritability is common across many DSM diagnoses, from MDD to ODD.

In contrast, other studies have taken a more transdiagnostic approach, examining shared effects or outcomes related to irritability. Stoddard and colleagues [11] conducted a multi-group study of children whose primary DSM diagnoses involved irritability, including participants with either primary BD, anxiety disorders, or meeting research criteria for severe mood dysregulation (SMD), defined as chronic, non-episodic irritability (the forerunner of DMDD). These participants did not differ on their self-reported irritability [11]. Furthermore, Cornacchio and colleagues [12] found a connection with irritability across childhood anxiety disorders, above and beyond a diagnosis of GAD. Studies comparing DMDD and ODD found that their symptom profiles co-occur and do not exist independently [13, 14]. The results of these transdiagnostic studies support research investigating a general psychopathology factor (p-factor) and the utility of a transdiagnostic approach to mental health. The p-factor integrates lower-order factors such as internalizing, externalizing, and thought disorders [15]. Studies have shown that the p-factor can be identified in children and adolescents, loading heavily on indicators of negative affect and dysregulation with predictive power of future functioning and mental health conditions [16, 17].

To help address knowledge gaps irritability in pediatric samples, our study goal was to recruit a sample of children and adolescents across the range of irritability and then evaluate how diagnostic categories impacted irritability measurement. This approach stands in contrast to narrower recruitment strategies based on single or aggregated DSM oriented diagnostic silos (e.g., Stoddard and colleagues [11]). We hypothesized that irritability would present itself similarly across different DSM diagnoses. We also sought to probe potential effects of age, hypothesizing younger children would display irritability more frequently than adolescents based on previous literature about behavioral presentations of irritability [18].

## Methods

### Participants

Children aged 8–16 years old were recruited from clinical and community settings to participate in two Institutional Review Board (IRB) approved studies with identical protocols investigating mechanisms of irritability. Studies were approved by Lifespan and Mass General Brigham IRBs and all procedures were performed in compliance with relevant laws and institutional guidelines. Informed consent and assent were obtained from parents and children/adolescents.

Children with any reported amount of irritability as measured by the Affective Reactivity Index (ARI) [19] were included, as indicated by parents on a phone screen. Total scores ranged from 0 to 12, with higher scores indicating greater irritability. To ensure a wide distribution of scores, participants were included based on tertiles of the ARI parent report of low (ARI score 0–4), medium (ARI score 5–8), and high irritability (ARI score 9–12). A more detailed description of the ARI is described below in measurement section.

Exclusion criteria were: (1) Wechsler Abbreviated Scale of Intelligence (WASI) [20] Full Scale IQ (FSIQ) < 80 due to possible misunderstanding of the study procedures; (2) active psychosis due to potential interference with understanding of study procedures; (3) non-removable metal including braces because the study also involved magnetic resonance imaging; and (4) color blindness due to potential misunderstanding of colored stimuli and feedback during study procedures.

### Measures

#### Demographic Information

Participants reported their age, race/ethnicity, and biological sex at their time of enrollment.

#### WASI

The WASI was administered to child participants as a measure of cognitive functioning to ensure understanding of study procedures. The WASI consists of two subtests, Vocabulary (31-item) and Matrix Reasoning (30-item), which yields the Full-Scale IQ-2 (FSIQ-2).

#### Kiddie Schedule for Affective Disorders and Schizophrenia Present and Lifetime Version for DSM-5 (KSADS) [21]

The KSADS is a semi-structured diagnostic clinical interview administered by trained graduate-level clinicians with established inter-rater reliability (kappa > 0.8) to child participants and their parents separately to evaluate current (most recent six months) and past (lifetime history up to six months ago) psychiatric diagnoses. No specific DSM-oriented KSADS diagnoses were required for participation.

#### ARI

The ARI is a six-item child (about self, ARI-C) and parent (about child, ARI-P) measure of irritability. Items such as “Is easily annoyed by others” were rated on a three-point scale (“*not true” [0]*,* “somewhat true”* [1], *and “certainly true”* [2]*).* ARI child report was obtained at the study visit. The measure has been found to be reliable in healthy and clinical youth samples across diagnoses [19, 22].

### Analytic plan

Mplus 8.6 [23] was used to implement all analyses. A confirmatory factor analysis (CFA) was used to assess whether the previously identified one-factor structure associated with both the ARI-P and ARI-C fit the data well. Overall model fit was evaluated using the comparative fit index (CFI), root mean square error of approximation (RMSEA), and standardized root mean square residual (SRMR). Cutoff values followed conventional standards, with good model fit indicated by a CFI≥0.95, RMSEA and SRMR≤0.06 [24]. Separate CFA models were established for the ARI-C and ARI-P, respectively.

Once good CFA model fit was established, multiple indicators multiple causes (MIMIC) modeling was used to assess for differential item functioning (DIF) among individual ARI-C/P items (see Wood and colleagues [25] for details on how MIMIC modeling can be used to define and assess for DIF). We assessed for DIF based on our hypothesized current KSADS diagnoses and child age. A test for DIF evaluates whether there are potential sources of item bias above and beyond mean differences. For example, a test for DIF evaluates whether the presentation of affective reactivity differs for children who are depressed (e.g., parents may not be as sensitive in detecting affective outbursts in youths with depression, even after accounting for differences in average level of irritability in depressed youths). Compared to traditional multiple group CFA that requires a separate model for each group, the MIMIC model incorporates grouping variables as covariates. This allows detection of group effects in cases with small sample sizes and can provide more stable estimates [26].

**Fig. 1:**
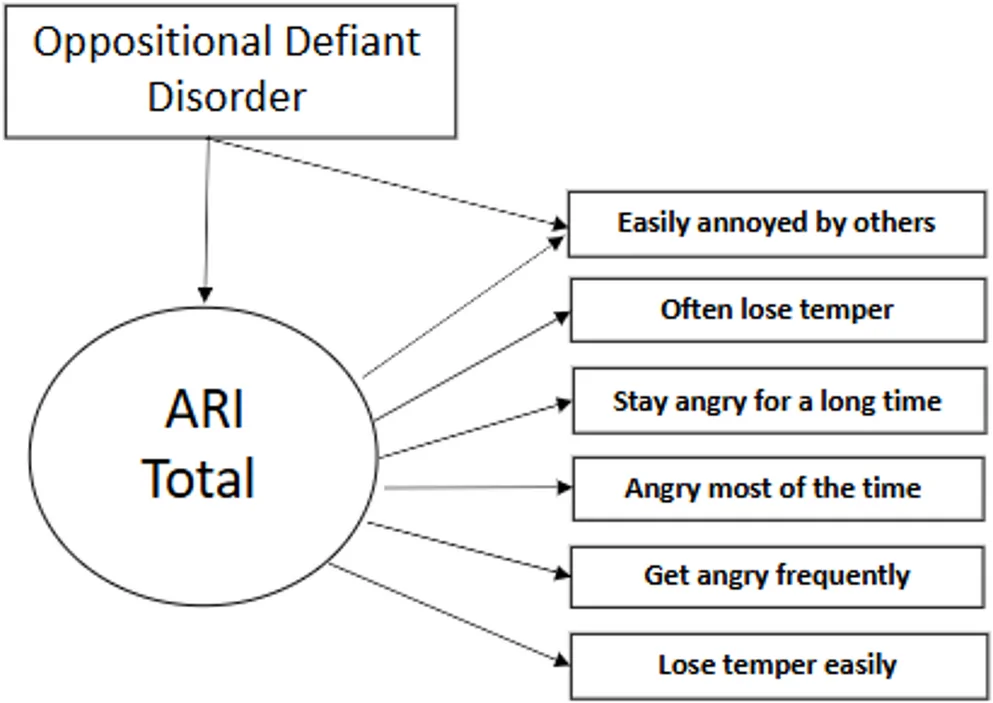
Example of MIMIC Invariance Test. Accessibility statement: Fig. 1 shows a circle that says ARI total with arrows that go to individual rectangles containing each of the ARI questions. An additional rectangle that says oppositional defiant disorder has an arrow going to the ARI total circle as well as the first rectangle containing one of the ARI questions

We evaluated whether ARI-C/P items exhibited DIF based on current child diagnosis, child age, and number of comorbid disorders (where we counted the number of comorbid conditions among all disorders evaluated for DIF; we only counted ADHD once). To achieve this end, the functioning of each individual covariate was assessed separately in the MIMIC model. As a result of this approach, multicollinearity does not impact results. We implemented this model by maintaining the regression of the latent factor explaining the ARI on each item, and then each symptom item was separately regressed on each of the covariates (see Fig. 1 for an example). If DIF is indicated for an item, then item endorsement is more/less likely than would be expected by affective reactivity alone (i.e., other unrelated factors are influencing item responses, such as perceptual biases). Diagnostic covariates represented discrete dichotomous influences on DIF, while the impact of child age and number of comorbid disorders on DIF was modeled through continuous variables. Hypothesis test *p*-values were evaluated for unstandardized estimates. As a post-hoc comparison, we also compared differences in level of DIF between all possible pairs of disorders. These tests were accomplished by regressing each ARI item on each disorder pair (e.g., ODD and MDD as two separate predictors) and then comparing the parameter estimates associated with each respective disorder via Wald tests of parameter constraints. Because these were post-hoc comparisons, we adjusted *p*-value comparisons to account for the false discovery rate (FDR) using procedures delineated by Benjamini and Hochberg [27].

To aid in interpretation, we reported results in terms of standardized betas. In the original CFA models, we evaluated R^2^ in terms of amount of variance explained in each item (also known as communalities in the factor analysis literature). To measure the magnitude of DIF in our MIMIC models, we evaluated the amount of variance in each item that was explained by each disorder (above and beyond the latent factor; referred to as ΔR^2^ in our results). According to criteria specified by Cohen [28], R^2^ values of 0.01, 0.09, and 0.25 correspond to small, medium, and large effect sizes respectively. All CFA-based models used mean- and variance-adjusted weighted least squares (WLSMV) estimation to accommodate the ordinal nature of ARI-C/P responses. We determined that a medium effect size would be the minimum effect size that would have clinical significance. Our a priori tests for DIF had over 95% power to detect a medium effect size. While exact power calculations for post-hoc adjustment by FDR depend on an unknown (number of true findings in the data), FDR is well suited to maintain a posteriori true effects at the original a priori alpha level [29]. While low sample size in subgroups can lead to imbalance that adversely affects power, this imbalance is built into the effect sizes that we used to conduct our power analysis.

### Missing data

Three child participants did not complete the ARI-C. Additionally, a single item response was missing for one child on the ARI-C and one parent on the ARI-P. Some children who provided ARI-C data did not provide information on their ethnicity (*n* = 11), and race (*n* = 5). Demographic summaries and percentage counts were reported based only on participants who provided this information. WLSMV estimation used in our CFA models addresses missing data through pairwise present covariance matrices.

## Results

**Fig. 2:**
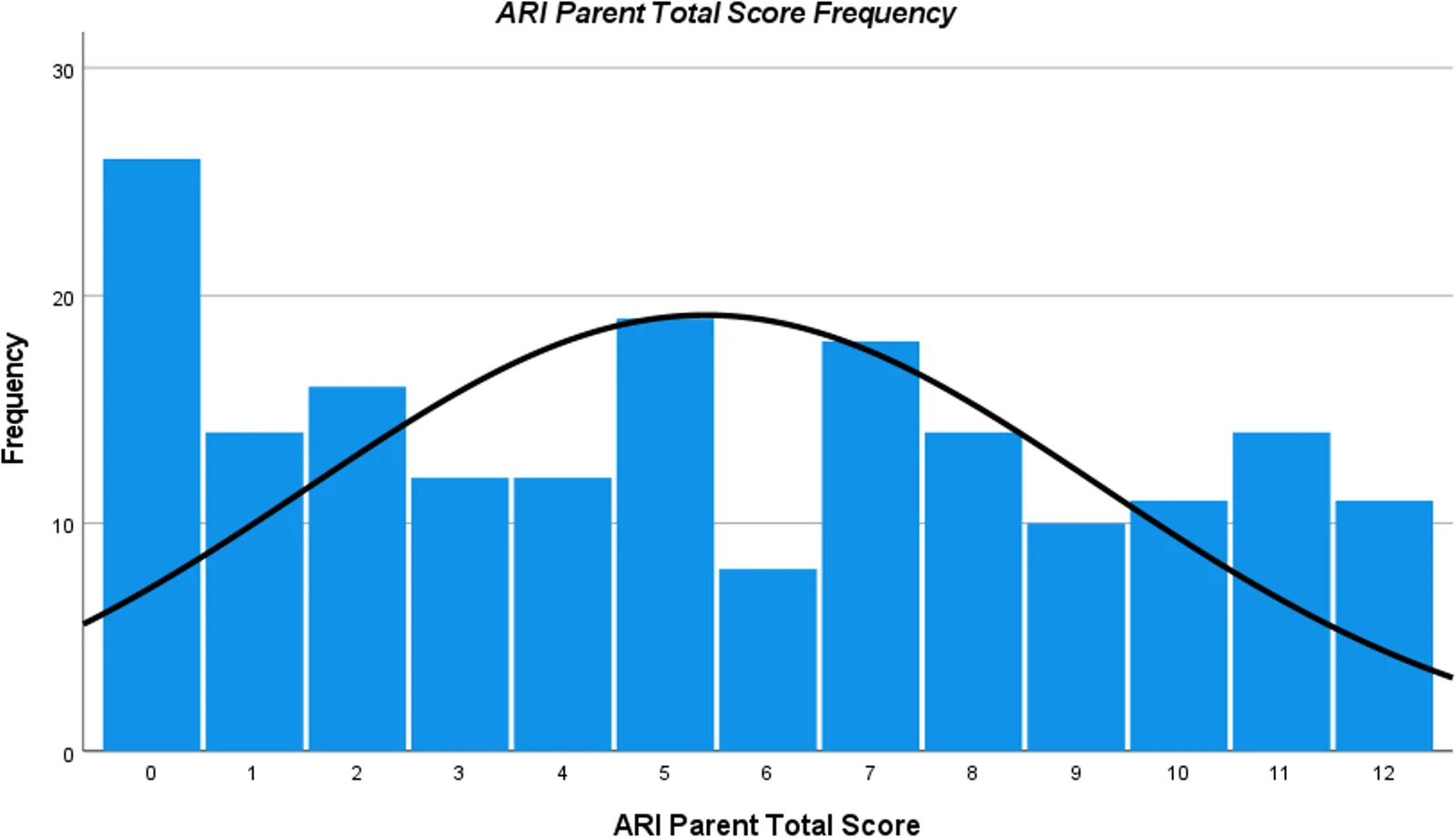
Distribution of ARI-P Scores. Accessibility statement: Fig. 2 is a bar graph of the frequency of the score on the ARI parent report. There is a curve to show the normal distribution

**Fig. 3:**
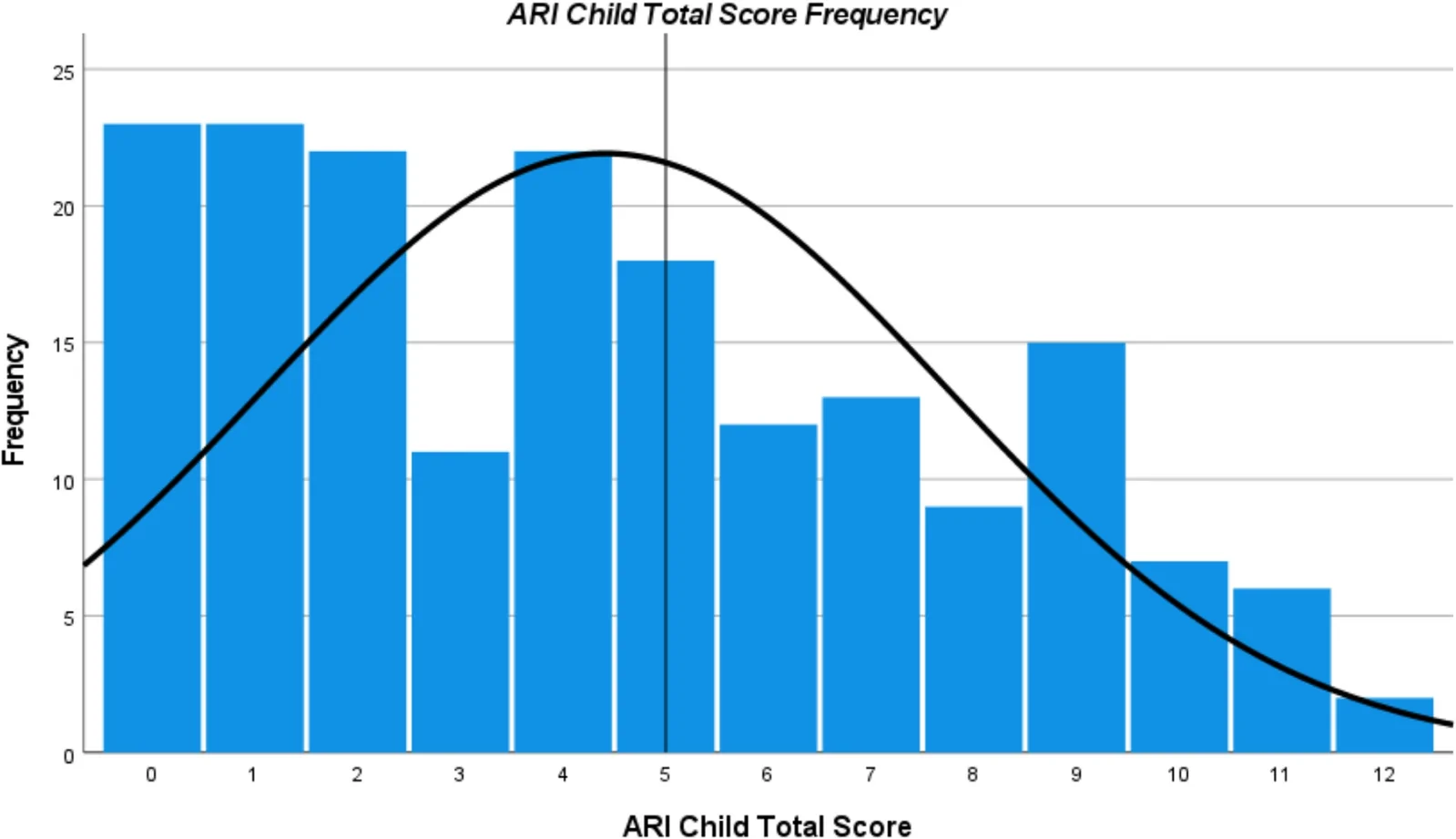
Distribution of ARI-C Scores. Accessibility statement: Fig. 3 shows a bar graph of the frequency of scores on the ARI child report. The curve of the normal distribution skews slightly left

We enrolled *n* = 186 participants with a mean age of 12.00 years (SD = 2.58; 44.0% assigned female at birth). 79.0% of participants identified as White, 8.8% as Black, 5.0% as Asian, and 7.2% identified their racial group as “other.” 12.6% of participating youth identified as Hispanic or Latino/a. Frequencies of diagnosis and comorbidity are listed in Table 1. As shown in Table 1, our most endorsed diagnoses were MDD, ODD, GAD, and ADHD. As these were our most prevalent diagnoses, we sought to analyze the relationship among these diagnoses and irritability. With respect to ADHD, we looked at ADHD inclusive of all subtypes as well as ADHD-combined subtype. The distribution of ARI-P and ARI-C scores are shown in Figs. 2 and 3.

**Table 1:** Disorder Prevalence and Comorbidity Rates in our Sample

	ND	ODD	MDD	GAD	ADHD^1^	ADHD^2^
ND	57(31.6)	-	-	-	-	-
ODD	-	65(34.9)	-	-	-	-
MDD	-	22(11.8)	54(29)			
GAD	-	23(12.4)	31(12.7)			
ADHD^1^	-	47(25.3)	25(13.4)			
ADHD^2^	-	32(17.2)	13(7)	15(8)	45(24.2)	45(24.2)

Disorder prevalence rates (N (%)) are displayed along the diagonal of the table and comorbidity rates (N (%)) are shown at the intersections of each disorder. *ND* no diagnosis, *ODD* oppositional defiant disorder, *MDD* major depressive disorder, *GAD* generalized anxiety disorder, *ADHD*^1^ attention-deficit/hyperactivity disorder any subtype, *ADHD*^*2*^ attention-deficit/hyperactivity disorder combined type. Less than 10% of our sample met diagnostic criteria for other diagnoses that include irritability as a symptom, including DMDD and Bipolar Disorder I and II

### Confirmatory factor analysis

A CFA to evaluate a fit of the single latent factor model for the ARI-C demonstrated an acceptable fit to the data (CFI>0.99, RMSEA=0.04, SRMR=0.03). A CFA to evaluate a fit of the single latent factor model for the ARI-P demonstrated acceptable to the data according to the CFI and SRMR but was slightly out of the target range for the RMSEA (CFI>0.99, RMSEA=0.07, SRMR=0.02). Upon review of potential reasons for model misfit, redundant item content was found that could adversely impact model fit on the ARI-P. Specifically, there was observed content overlap between ARI-P items 2 (often lose temper) and 6 (lose temper easily) that could lead to residual correlation. Once this residual correlation was incorporated into the model, the ARI-P demonstrated an acceptable fit to the data across all fit indices considered (CFI>0.99, RMSEA=0.06, SRMR=0.02).

Given that this model’s modifications did not have implications for ARI-P factor structure or clinical scoring, we used this modified model in all subsequent MIMIC models. Parameter estimates for all CFA models are displayed in Tables 2 and 3. We also added beta values associated with paths from covariates to latent variables in Supplemental Tables S1 (ARI-C) and S2 (ARI-P).

**Table 2:** CFA and MIMIC Results for the ARI-P

Initial CFA		MIMIC – ARI-C ΔR^2^						
Item	R^2^	ODD	MDD	GAD	ADHD^1^	ADHD^2^	Age	Number Disorders
1. Easily annoyed by others	0.79	< 0.01	0.01*	0.01*	< 0.01	< 0.01	< 0.01	< 0.01
2. Often lose temper	0.82	< 0.01	0.01*	0.01	< 0.01	< 0.01	0.01	0.01*
3. Stay angry for a long time	0.59	< 0.01	< 0.01	< 0.01	< 0.01	< 0.01	< 0.01	< 0.01
4. Angry most of the time	0.81	< 0.01	< 0.01	< 0.01	< 0.01	0.01	< 0.01	< 0.01
5. Get angry frequently	0.93	0.01	< 0.01	< 0.01	< 0.01	< 0.01	< 0.01	0.01
6. Lose temper easily	0.83	< 0.01	0.02	< 0.01	< 0.01	0.01*	0.03*	< 0.01

R^2^ for item reflects amount of variance explained in each item explained by the latent variable (i.e., communality). ΔR2 reflects amount of variance explained by each disorder above and beyond the latent variable (i.e., effect size for observed invariance). Asterisk reflects statistically significant DIF at the *p*<.05 level. “Number Disorders” = number of comorbid disorders (range of 0–4 in sample). *ADHD*^1^ attention-deficit/hyperactivity disorder any subtype, *ADHD*^2^ attention-deficit/hyperactivity disorder combined type

**Table 3:** CFA and MIMIC Results for the ARI-C

Initial CFA		MIMIC – ARI-C ΔR^2^						
Item	R^2^	ODD	MDD	GAD	ADHD^1^	ADHD^2^	Age	Number Disorders
1. Easily annoyed by others	0.52	< 0.01	< 0.01	0.01	< 0.01	< 0.01	0.01	0.01
2. Often lose temper	0.80	< 0.01	< 0.01	< 0.01	0.01	0.03*	< 0.01	< 0.01
3. Stay angry for a long time	0.50	< 0.01	< 0.01	< 0.01	< 0.01	< 0.01	< 0.01	< 0.01
4. Angry most of the time	0.56	< 0.01	< 0.01	< 0.01	< 0.01	< 0.01	0.02	< 0.01
5. Get angry frequently	0.72	< 0.01	< 0.01	< 0.01	< 0.01	< 0.01	0.01	< 0.01
6. Lose temper easily	0.85	< 0.01	< 0.01	< 0.01	< 0.01	0.01	< 0.01	< 0.01

R^2^ for item reflects amount of variance explained in each item explained by the latent variable (i.e., communality). ΔR2 reflects amount of variance explained by each disorder above and beyond the latent variable (i.e., effect size for observed invariance). Asterisk reflects statistically significant DIF at the *p*<.05 level. “Number Disorders” = number of comorbid disorders (range of 0–4 in sample). *ADHD* ^1^ attention-deficit/hyperactivity disorder any subtype, *ADHD* ^2^ attention-deficit/hyperactivity disorder combined type

### Differential item functioning for individual ARI-C/P items

Results for MIMIC models for the ARI-P can be found in Table 2. Statistically significant effects of DIF were observed for MDD on items 1 (“easily annoyed by others”; *b* = 0.11, *p*=.046, ΔR2=0.01) and 2 (“often lose temper”; *b*=−0.10, *p*=.033, ΔR2=0.01), for GAD on item 1 (“easily annoyed by others”; *b* = 0.14, *p*=.026, ΔR2=0.01), ADHD – Combined type on item 6 (“lose temper easily”; *b* = 0.12, *p*=.018, ΔR2=0.01), and for number of comorbid disorders on item 2 (*b*=−0.11, *p*=.036, ΔR2=0.01). Additionally, DIF was statistically significant for age on item 6 (*b*=−0.13, *p*=.020, ΔR2=0.03). All observed DIF was in the small effect size range. No significant differences were observed in between-disorder comparisons of DIF levels on the ARI-P after accounting for the FDR.

Results for MIMIC models for the ARI-C can be found in Table 3. The only statistically significant effect observed was for item 2 (“often lose temper”) on the ARI-C for ADHD-Combined Type (*b* = 0.15, *p*=.010, ΔR^2^=0.03), and this was also in the small effect size range. No significant differences were observed in between-disorder comparisons of DIF levels on the ARI-C after accounting for the FDR.

## Discussion

Our study sought to examine diagnostic associations of irritability in a transdiagnostic sample recruited across the range of irritability rather than from specific DSM disorders. Our primary finding was that irritability, measured by the ARI, was reported similarly across diagnoses, regardless of whether the participant had diagnoses of MDD, ODD, GAD, or ADHD, supporting previous research that the ARI is relatively stable across conditions [1, 19]. Additionally, we found there were no large differences in how irritability manifested across ages 8–16. Our study limitations include self-report bias, low participant numbers across other DSM diagnoses, limited age range, and limited racial and ethnic diversity in our sample. Nevertheless, our findings have important implications for how irritability is assessed and important considerations for treatment and future research.

Overall, we found minimal differences in how irritability was manifested across different primary diagnoses associated with irritability in both parent and child reports. While there were statistically significant differences in some parent reports of irritability, the small effect sizes suggest that the differences were negligible in magnitude. Specifically, in our transdiagnostic sample, we found statistically significant effects of DIF in MIMIC models for the ARI-P in MDD, GAD, and ADHD-Combined type, but none of these effects explained more than 1% in item variance. Previous research has indicated differences in parent perception of irritability when compared to child report, especially for internalizing versus externalizing disorders [11, 30, 31]. For example, our results are similar to those of Stoddard and colleagues [11] who found that while child self-report measures of irritability did not significantly differ between the anxiety, BD, and SMD groups, they did find significant between-group differences in parent report measures about their child’s irritability. Furthermore, our results align with studies showing how common irritability is endorsed in children whose mental health conditions do not involve irritability as an explicit diagnostic criterion. Evans and colleagues [30] looked at 237 children ages 3–18 presenting for outpatient treatment and evaluated irritability across diagnoses, such as ADHD, MDD, ODD, and learning disorders. Results showed that while ARI-P and ARI-C scores were both significantly related to measures of inattention, hyperactivity/impulsivity, executive dysfunction, and aggression, only the ARI-C was significantly related to anxiety and depression. Additionally, only the ARI-P was significant for peer problems. Taken as a whole, our results contribute to a body of research that supports irritability as a transdiagnostic symptom in childhood and adolescence.

Our study emphasizes the importance of assessing irritability in children and teenagers, regardless of whether it is a criterion for a specific diagnosis. Our results indicate that irritability manifests similarly in different internalizing and externalizing disorders, including ADHD for which it is not a diagnostic symptom in the DSM 5 [2]. The study supports previous research demonstrating the pervasiveness of irritability in pediatric psychopathology [11, 32, 33]. It is important that clinicians assess irritability regardless of the identified primary diagnosis, as well as conceptualizing it in terms of both mood and behavior to improve long term outcomes [32]. For example, chronic childhood irritability is shown to be associated with depressive disorders, GAD, ODD, suicidal thoughts and behaviors, and poorer educational attainment across the lifespan, while episodic irritability is associated with later development of GAD and mania [33–37]. Therefore, incorporating assessments of irritability, and treating accordingly, is essential for overall functioning.

Notably, our study found age-based associations with irritability reported by parents, but not children themselves. Consistent with previous research, we did identify “lose temper easily” on the ARI-P to have statistically significant variance based on child age, with lower rates of item endorsement found for older youths after accounting for overall levels of irritability. This result could be explained by a typical reduction in irritability as children age, potentially related to the growth of emotion regulation skills, including cognitive and language development, coinciding with development of the prefrontal cortex into the mid-20s [38]. The delayed cognitive development in younger children could explain the difference in perception of behavior from their parents as they may lack insight into their own behaviors [38]. Behavioral expressions of anger commonly seen in toddlerhood tend to decrease from preschool to school-aged children with further improvement in behavioral inhibition and anger regulation in adolescents [18, 38–40]. Our results, in concert with prior findings, suggest the opportunity for assessment and treatment efforts for irritability to be tailored to fit the developmental stage, including cognitive and language development, of the child.

### Limitations and future directions

Limitations of our study that should be noted include self-report bias, distribution of DSM disorders, age range, and ethno-racial diversity. First, our results are subject to self-report bias given our use of the ARI-C/P and KSADS self-report. However, we worked to mitigate self-report bias by obtaining the same information from both children and parents, and our analyses were explicitly designed to assess for rater bias. Second, given our transdiagnostic sample was recruited across the range of irritability, rather than explicitly recruited to produce a minimum number of participants with specific DSM diagnoses, we did not have a large enough sample of other disorders beyond MDD, GAD, ADHD, and ODD to use as a basis for measurement invariance tests. For example, we had insufficient numbers of participants with DMDD, fewer than 10%, to examine specific correlates. However, we did not have a sampling bias for DMDD, or any other DSM diagnoses, because DSM diagnoses were not part of our recruitment strategy or inclusion criteria. This suggests a need for additional studies comparing large samples of children whose primary DSM diagnosis involves irritability, as well as larger transdiagnostic samples, to see if our irritability results generalize beyond our most prevalent diagnoses. Third, our sample looked at elementary school aged through high school aged children. Therefore, our age-related ARI-P results may not be generalizable to preschool children and/or older adolescents/transition age youths. Encompassing a wider age range in future research could help to further understand irritability manifestations across development. Last, while we had a range of reported ethnicities, most of our sample identified as white. In attempts to mitigate this, we sought to recruit participants through community health events, hospital healthcare records at various levels of care and specialties, and community Facebook pages. Future studies should assess reporting and manifestations of irritability across diverse racial and ethnic populations to help improve diagnosis and treatment.

As our findings support other studies indicating that irritability present similarly various diagnosis, it raises the question of how best to treat irritability. Some have suggested it is best to treat the primary diagnoses associated with irritability, such as MDD, for which there are disorder-specific treatment manuals and medication protocols [32]. In contrast, another approach that aligns with the idea that irritability is a transdiagnostic problem would be to treat the irritability, regardless of primary diagnoses. For example, the Unified Protocol (UP)’s transdiagnostic approach has shown strong treatment effects, with greater improvement in irritability, along with sadness, anxiety, and cognitive flexibility in children and adults [41–43]. Current research has shown support for both disorder specific and a UP approach to treating irritability in children [43–46]. Given the findings from our transdiagnostic study, further research on the UP for children and adolescents and the impact on irritability is needed to establish efficacy of these treatments. This could decrease the burden on children and families struggling with varying levels of irritability and improve long-term functioning.

## Conclusion

Our results suggest that irritability manifests itself similarly in youth across different diagnostic categories and age ranges. Our study supports the use of the ARI in transdiagnostic research for children, helping to further previous research that has worked to elucidate similarities and differences among diagnoses and presentations. Future research in this area using behavioral and imaging tasks could support transdiagnostic neural and behavioral markers. As irritability becomes more clearly understood across development and psychopathology, this could lead to improved assessment and treatment of one of the most impairing psychological symptoms.

## Supplementary Information

Below is the link to the electronic supplementary material.

Supplementary Material 1 (DOCX 20.5 KB)

## Data Availability

We welcome inquiries from investigators who would want to collaborate with our data. The corresponding author can be contacted with any interest.
