# Supplementary material for: Diagnostic- and age-based measurement invariance of irritability in youths
Source: Eur Child Adolesc Psychiatry. 2026 May 9;35(9):2759–68. doi: 10.1007/s00787-026-03037-z (PMC13695628; doi:10.1007/s00787-026-03037-z)
Supplement: Supplementary file 1 [file 787_2026_3037_MOESM1_ESM.docx]

Supplementary Table S1. Standardized Beta Values Associated with Paths from Covariates to Latent Variables in our ARI-C MIMIC Models.

|  | ODD | MDD | GAD | ADHD^1^ | ADHD^2^ | Age | Number Disorders |
| --- | --- | --- | --- | --- | --- | --- | --- |
| 1. Easily annoyed by others | 0.303 | 0.307 | 0.181 | 0.219 | 0.166 | <.001 | 0.394 |
| 2. Often lose temper | 0.310 | 0.326 | 0.203 | 0.208 | 0.134 | 0.034 | 0.407 |
| 3. Stay angry for a long time | 0.301 | 0.322 | 0.193 | 0.231 | 0.171 | 0.026 | 0.407 |
| 4. Angry most of the time | 0.294 | 0.321 | 0.205 | 0.233 | 0.177 | 0.038 | 0.408 |
| 5. Get angry frequently | 0.321 | 0.324 | 0.200 | 0.237 | 0.176 | 0.001 | 0.421 |
| 6. Lose temper easily | 0.320 | 0.316 | 0.209 | 0.233 | 0.189 | 0.015 | 0.420 |

*Note.* “Number Disorders” = number of comorbid disorders (range of 0-4 in sample). ADHD^1^ = attention-deficit/hyperactivity disorder any subtype, ADHD^2^ = attention-deficit/hyperactivity disorder combined type.

Supplementary Table S2. Standardized Beta Values Associated with Paths from Covariates to Latent Variables in our ARI-P MIMIC Models.

|  | ODD | MDD | GAD | ADHD^1^ | ADHD^2^ | Age | Number Disorders |
| --- | --- | --- | --- | --- | --- | --- | --- |
| 1. Easily annoyed by others | 0.537 | 0.304 | 0.189 | 0.426 | 0.416 | -0.134 | 0.570 |
| 2. Often lose temper | 0.537 | 0.339 | 0.233 | 0.428 | 0.415 | -0.105 | 0.599 |
| 3. Stay angry for a long time | 0.538 | 0.322 | 0.212 | 0.431 | 0.415 | -0.128 | 0.585 |
| 4. Angry most of the time | 0.520 | 0.314 | 0.209 | 0.431 | 0.427 | -0.129 | 0.574 |
| 5. Get angry frequently | 0.549 | 0.319 | 0.220 | 0.431 | 0.412 | -0.126 | 0.592 |
| 6. Lose temper easily | 0.522 | 0.337 | 0.229 | 0.415 | 0.393 | -0.097 | 0.583 |

*Note.* “Number Disorders” = number of comorbid disorders (range of 0-4 in sample). ADHD^1^ = attention-deficit/hyperactivity disorder any subtype, ADHD^2^ = attention-deficit/hyperactivity disorder combined type.
